# A Retrospective Survey of Rodent-borne Viruses in Rural Populations of Brazilian Amazon

**DOI:** 10.1590/0037-8682-0511-2019

**Published:** 2020-06-22

**Authors:** Jorlan Fernandes, Thayssa Alves Coelho, Renata Carvalho de Oliveira, Alexandro Guterres, Claudia Lamarca Vitral, Bernardo Rodrigues Teixeira, Fernando de Oliveira Santos, Jaqueline Mendes de Oliveira, Mônica da Silva-Nunes, Marco Aurélio Pereira Horta, Silvana C. Levis, Marcelo Urbano Ferreira, Elba Regina Sampaio de Lemos

**Affiliations:** 1 Fundação Oswaldo Cruz, Instituto Oswaldo Cruz, Laboratório de Hantaviroses e Rickettsioses, Rio de Janeiro, RJ, Brasil.; 2 Universidade Federal Fluminense, Instituto Biomédico, Niterói, RJ, Brasil.; 3 Fundação Oswaldo Cruz, Instituto Oswaldo Cruz, Laboratório de Biologia e Parasitologia de Mamíferos Silvestres Reservatórios, Rio de Janeiro, RJ, Brasil.; 4 Fundação Oswaldo Cruz, Instituto Oswaldo Cruz, Laboratório de Desenvolvimento Tecnológico em Virologia, Rio de Janeiro, RJ, Brasil.; 5 Universidade Federal do Acre, Centro de Ciências da Saúde, Rio Branco, AC, Brasil.; 6 Fundação Oswaldo Cruz, Instituto Oswaldo Cruz, Rio de Janeiro, RJ, Brasil.; 7 Instituto Nacional de Enfermedades Virales Humanas, Pergamino, Argentina.; 8 Universidade de São Paulo, Instituto de Ciências Biomédicas, Departmento de Parasitologia, São Paulo, SP, Brasil.

**Keywords:** Zoonoses, Brazilian Amazonian region, Mammarenavirus, Hantavirus, Rodent-borne disease

## Abstract

**INTRODUCTION:**

The Amazon tropical rainforest has the most dense and diverse ecosystem worldwide. A few studies have addressed rodent-borne diseases as potential hazards to humans in this region.

**METHODS:**

A retrospective survey was conducted using enzyme-linked immunosorbent assay for detecting mammarenavirus and orthohantavirus antibodies in 206 samples collected from rural settlers of the Brazilian Western Amazonian region.

**RESULTS:**

Six (2.91%) individuals in the age group of 16 to 36 years were found to possess antibodies against mammarenavirus.

**CONCLUSION:**

Evidence of previous exposure to mammarenavirus in the rural population points to its silent circulation in this region.

The Amazon region (Amazon River Basin) is a vast territory that encompasses parts of nine South American countries, including a large portion of Brazil. This region has the largest tropical rainforest in the world with a climate that is characterized by high temperature and humidity levels, copious rainfalls, and the most dense and diverse ecosystem worldwide^1^. 

Demographic density in the Brazilian Amazonian region is low (4.7 persons/km^2^), and many areas are nearly bereft of healthcare facilities. Paradoxically, an intense urbanization process has been taking place in this region in the last few years^1^ ^,^ ^2^. Since the 1970s, the Brazilian government has been creating rural settlements in the Amazon, and in this region, agriculture and farming are the two main economic activities conducted for livelihood^2^. 

The conditions in the Brazilian Amazonian region are favorable for the transmission of numerous tropical infectious agents, which pose particular risks to the health of the population as they are exposed to hazardous housing and working conditions^2^. Recently, a new mammarenavirus was identified in rodents that were captured in Acre state, Brazil. This virus seems to be the result of reassortment between two distinct mammarenavirus clades and is the first known mammarenavirus identified from this region^3^. Only a few studies have addressed rodent-borne diseases, such as mammarenavirus and orthohantavirus infections, as potential hazards to humans in this region, although the human and rodent infections, mostly related to orthohantavirus, have previously been reported in the Northern region of Brazil^3^ ^-^ ^8^. In the present study, we aimed to retrospectively examine the seroprevalence of mammarenavirus and orthohantavirus in a rural population located in the Brazilian Western Amazonian region.

The surveyed area, known as “Ramal do Granada” (9^o^41’S-9^o^49’S, 67^o^05’W-67^o^07’W), is a sparsely populated rubber tapper settlement in the Acrelândia municipality of the Acre state that is a part of the Pedro Peixoto Agricultural Settlement Project. The Ramal do Granada has a linear extension of approximately 30 km, and includes households along an unpaved road with an economy based on agriculture, and mainly the livestock. Blood samples were collected during a cross-sectional survey in 2004 and were stored in a -20 °C freezer^9^. In total, 206 serum samples were subjected to serological analysis using the enzyme-linked immunosorbent assay (ELISA), according to a previously published protocol^10^. Antigens were derived from the Vero C76 cells (ATCC^®^ CRL-1587™) infected with Junín mammarenavirus (Clade B New World mammarenaviruses) or Maciel orthohantavirus (Andes orthohantavirus group). The cut-off (0.2) was determined by estimating the mean optical densities (OD) of the negative controls with three standard deviations at 1:100 dilution^10^. The results were used to analyze the data together with the information that was gathered through a structured questionnaire^9^. The protocols implemented in this study were approved by the Research Ethics Committee for Experimentation in Human Beings of the Instituto de Ciências Biomédicas, Universidade de São Paulo as reported previously^9^, and by the Fundação Oswaldo Cruz/Instituto Oswaldo Cruz under the approval number CAAE 61629416.2.1001.5248.

Data regarding the use of land of the Acrelândia municipality was obtained from MapBiomas v.3.1 (http://www.mapbiomas.org/) and was used to construct the comparative maps between 2004 and the more recently available data from 2016. Geoprocessing was performed using the Quantum Gis^®^ program (QGIS Development Team, 2017)^11^. We used the resultant report of QGIS to calculate the extension (km²) covered by the forest and modified (pasture/agriculture) areas between 2004 and 2016. Aiming to test the relationship between social factors, including time of residence, house material, type of sewage coverage, presence of pets, profession, sex, and age as shown in Table 1, and the outcome variable (positivity for anti-mammarenavirus and /or anti-orthohantavirus IgG antibodies), Chi-squared tests (X^2^) were performed with statistical significance defined at *p-value* < 0.05. Data analysis was performed using the statistical package of R Studio (version 1.1.463).

The age of the 206 individuals that participated in this study was from a few months to 90 years (24.43 median years). Most of the participants were female 51.5 % (106), farmers 28.6 % (59), and students 28.2 % (58) who were living in the wood houses 82.5 % (170), and had no access to sewerage and garbage collection. Variables are presented in Table 1.

**TABLE 1:** Mammarenavirus seropositivity and Chi-square test (*p-value*) as per the categorical variable in Ramal do Granada population, Acre state, Brazil.

Categorical variable	Number of subjects (%)	Seropositivity (%) (95% CI)	X^2^ (p-value)
**Age**			0.10
<12	62 (30.1)	0 (0.0)	
13-17	25 (12.1)	2 (8.0) (2.2-25.0)	
18-30	51 (24.8)	3 (5.9) (2.0-15.9)	
>31	68 (33.0)	1 (1.5) (0.3-7.9)	
**Sex**			0.44
Women	106 (51.5)	4 (3.8) (1.5-9.3)	
Men	100 (48.5)	2(2.0) (0.6-7.0)	
**Time of residence in the area**			0.38
< 5 years	69 (33.5)	2 (2.9) (0.8-10.0)	
6 - 15 years	80 (38.8)	1 (1.2) (0.2-6.7)	
> 16 years	57 (27.7)	3 (5.3) (1.8-14.4)	
**House material**			0.52
Brick	15 (7.3)	0 (0.0)	
Straw	21 (10.2)	0 (0.0)	
Wood	170 (82.5)	6 (3.5) (1.6-7.5)	
**Sewage**			0.52
Septic tank	22 (10.7)	0 (0.0)	
Open trench	170 (82.5)	6 (3.5) (1.6-7.5)	
Other	14 (6.8)	0 (0.0)	
**Main activity developed on the property**			0.94
Agriculture	44 (21.4)	1 (2.3) (0.4-11.8)	
Cattle raising	134 (65.0)	4 (3.0) (1.2-7.4)	
None	28 (13.6)	1 (3.6) (0.6-17.7)	
**Pets on the property**			0.45
No	17 (8.3)	0 (0.0)	
Yes	189 (91.7)	6 (3.2) (1.5-6.8)	
**Hunting and fishing**			0.35
No	70 (34.5)	1 (1.4) (0.3-7.7)	
Yes	133 (65.5)	5 (3.8) (1.6-8.5)	
**Profession**			0.57
Farmer	59 (28.6)	1 (1.7) (0.3-9.0)	
Housekeeping	35 (17.0)	2 (5.7) (1.6-18.6)	
Student	58 (28.2)	2 (3.4) (1.0-11.7)	
Education worker	12 (5.8)	1 (8.3) (1.5-35.4)	
Other school activities^*a*^	28 (13.6)	0 (0.0)	
Other^*b*^	14 (6.8)	0 (0.0)	

a Includes teachers and school staff (cleaners and cooks). ^b^ Including all occupations with fewer than three mentions.

None of these individuals exhibited the presence of antibodies against orthohantavirus. Low prevalence ratios of 1.1 % and 0.8 % in the Amazon basin rural population from Peru and Brazil, respectively, have been reported previously^4^ ^,^ ^12^. The low seroprevalence to orthohantavirus observed in this study could be probably due to low agricultural activities in these particular region and the main activity being carried out was cattle raising (Table 1). Evidence of orthohantavirus circulation in wild rodent species, including *Oligoryzomys microtis* and *Proechimys cuvieri*, have been recorded in Acre state, although no orthohantavirus pulmonary syndrome cases among the individuals have been reported yet^8^. Therefore, more studies including large number of individuals are required in Acre state in order to better evaluate the impact of orthohantavirus infections in humans and rodents.

Mammarenavirus antibodies were detected in six young and adult individuals (age between 16 to 36 years), with an overall seroprevalence rate of 2.91 %. The seropositivity rate was slightly higher in females (3.8 %) than in males (2.0 %). It is noteworthy that five of the six individuals with antibodies against mammarenavirus mentioned that they performed hunting and fishing for their livelihood. No significant association was found between mammarenavirus seropositivity and work activities or other variables (Table 1), probably because of the low seroprevalence ratio, however, the prevalence observed was higher than those found in other previous studies that were conducted in Brazil and Colombia^6^ ^,^ ^10^ ^,^ ^13^. 

To date, only a few cases of Brazilian hemorrhagic fever, which is caused by the Sabiá mammarenavirus, has been described in São Paulo region, southeastern Brazil^6^ ^,^ ^14^. However, five mammarenaviruses have been identified in rodents during the surveys that were conducted in the Brazilian Amazonian region and are listed as follows: (1) Amaparí virus (*Neacomys guianae*); (2) Cupixi virus (*Hylaeamys megacephalus*); (3) Flexal virus (unidentified oryzomyini); (4) Latino virus (*Calomys callidus*); (5) the most recently identified Xapuri virus (*Neacomys musseri*), demonstrating the potential for mammarenavirus emergence in this region^3^ ^,^ ^7^ ^,^ ^14^.

The area under study has a history of urbanization similar to the other regions of the Amazon basin, which started with the rubber boom in the early 20^th^ century followed by other extractive activities, such as mining and lumber industries^16^. In 2004, the Acrelândia municipality had 983.3008 km² as the forested area (62.8 % of the total municipality area) and 580.2529 km² as the pasture and agriculture area (37.1 %). In 2016, a decrease in the forested area and an increase in the modified area were observed, leading to the existence of 657.6028 km² of forested area (42.0 %) and 901.6469 km² of pasture and agriculture area (57.6 %), as shown in Figure 1. Over the last several decades, agriculture has been the main factor that is responsible for the continued deforestation in the Pedro Peixoto settlement (Figure 1), possibly due to the poor technology applied for farming. This probably led to an increase in the contact between humans and wildlife, and a higher probability of the emergence of infectious diseases in this region^2^ ^,^ ^15^ ^,^ ^16^. As reported in the previous studies, the high prevalence of zoonotic infections associated with Ramal do Granada inhabitants is suggestive of the fact that they are previously exposed to a wide variety of pathogens^9^ ^,^ ^15^. Many of these diseases, such as dengue, yellow fever, and malaria, are responsible for hundreds of cases, and could be easily misdiagnosed as mammarenavirus cases, especially because of the lack of healthcare services and healthcare professional training and distribution, even with the current advances in the Brazilian public health care system^1^. Similar ecological and economic scenarios were reported during the emergence of Venezuelan hemorrhagic fever that is caused by Guanarito virus. This virus was first recognized during a dengue fever outbreak in Venezuela when the health authorities and physicians noticed “atypical” dengue hemorrhagic cases that continued to occur in the Portuguesa state, although these cases have decreased all over the country with time^6^ ^,^ ^14^.

**FIGURE 1:**
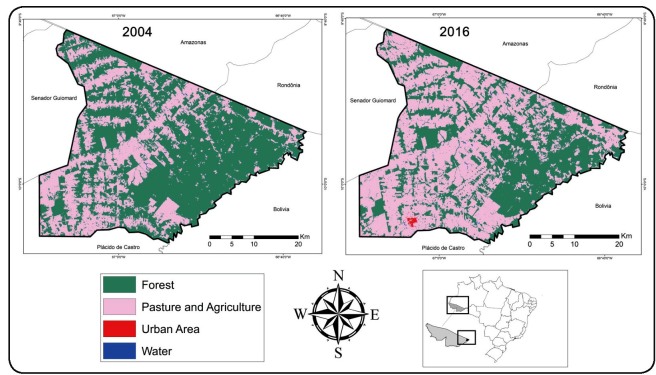
Comparative maps depicting the use of land between 2004 and 2016 in the Acrelândia municipality, Acre state, Brazil.

Historically, the Northern and Northeastern regions of Brazil, which include most of the Amazon River basin, exhibits the highest social inequalities and prevalence of infectious diseases^1^ ^,^ ^2^. Although additional investigations are required to be conducted, the identification of evidence of exposure to mammarenavirus infection in the Amazon basin indicates the occurrence of silent circulation of these emergent viruses in this region, and urges to include these viruses in the syndromic surveillance approach for febrile hemorrhagic diseases. Further studies in this region will help to better understand the mechanism by which the Amazon rural population is exposed to these zoonotic agents, and to characterize the circulating mammarenavirus species responsible for the human infections. 
